# Zearalenone regulates key factors of the Kelch-like erythroid cell-derived protein with CNC homology-associated protein 1–nuclear factor erythroid 2-related factor 2 signaling pathway in duodenum of post-weaning gilts

**DOI:** 10.5713/ajas.20.0384

**Published:** 2020-10-13

**Authors:** Qun Cheng, Shu zhen Jiang, Li bo Huang, Wei ren Yang, Zai bin Yang

**Affiliations:** 1 Shandong Provincial Key Laboratory of Animal Biotechnology and Disease Control and Prevention, Department of Animal Sciences and Technology, Shandong Agricultural University, Taian, Shandong 271018, China

**Keywords:** Antioxidant, Duodenum, Keap1-Nrf2 Signaling Pathway, Oxidative Stress, Zearalenone

## Abstract

**Objective:**

This study explored the mechanism of the Kelch-like erythroid cell-derived protein with CNC homology-associated protein 1 (Keap1)–nuclear factor erythroid 2-related factor 2 (Nrf2) signaling pathway under conditions of zearalenone (ZEA)-induced oxidative stress in the duodenum of post-weaning gilts.

**Methods:**

Forty post-weaning gilts were randomly allocated to four groups and fed diets supplemented with 0, 0.5, 1.0, or 1.5 mg/kg ZEA.

**Results:**

The results showed significant reductions in the activity of the antioxidant enzymes total superoxide dismutase and glutathione peroxidase and increases the malondialdehyde content with increasing concentrations of dietary ZEA. Immunohistochemical analysis supported these findings by showing a significantly increased expression of Nrf2 and glutathione peroxidase 1 (GPX1) with increasing concentrations of ZEA. The relative mRNA and protein expression of Nrf2, GPX1 increased linearly (p<0.05) and quadratically (p<0.05), which was consistent with the immunohistochemical results. The relative mRNA expression of Keap1 decreased linearly (p<0.05) and quadratically (p<0.05) in the duodenum as the ZEA concentration increased in the diet. The relative mRNA expression of modifier subunit of glutamate-cysteine ligase (GCLM) increased quadratically (p<0.05) in all ZEA treatment groups and the relative mRNA expression of quinone oxidoreductase 1 (NQO1) catalytic subunit of glutamate-cysteine ligase decreased linearly (p<0.05) and quadratically (p<0.05) in the ZEA1.0 group and ZEA1.5 group. The relative protein expression of Keap1 and GCLM decreased quadratically (p<0.05) in the duodenum as the ZEA concentration increased in the diet, respectively. The relative protein expression of NQO1 increased linearly (p<0.05) and quadratically (p<0.05) in all ZEA treatment groups in the duodenum.

**Conclusion:**

These findings suggest that ZEA regulates the expression of key factors of the Keap1-Nrf2 signaling pathway in the duodenum, which enables resistance to ZEA-induced oxidative stress. Further studies are needed to examine the effects of ZEA induced oxidative stress on other tissues and organs in post-weaning gilts.

## INTRODUCTION

Zearalenone (ZEA) is a contaminant widely found in cereals and compound feeds that exhibits latent toxicity. This can cause significant economic losses due to reductions in animal production, as well as adversely affecting public health and animal welfare [1,2]. Zearalenone, 6-(10-hydroxy-6-oxo-trans-1-undecenyl)-β-resorcyclic acid lactone, is a nonsteroidal estrogen mycotoxin produced by *Fusarium* fungi [3,4]. The structure of ZEA is similar to that of estradiol, which can bind to estrogen receptors in 17 β-estradiol-competitive target cells, resulting in reproductive dysfunction and decreased reproductive capacity [5,6]. Different levels of ZEA cause different reactions in animals, and can result in reduced productivity, oxidative damage to organs and tissues, immune stress, reduced reproductive performance, and may even lead to acute death [7–9]. According to recent reports, the toxic effects of ZEA are not limited to estrogen-caused reproductive toxicity, but include other mechanisms that target general cells and tissues [10,11]. The oxidative stress caused by the accumulation of reactive oxygen species (ROS) and the regulation of antioxidant enzymes may be one of the main mechanisms of *in vivo* and *in vitro* ZEA toxicity [12].

It has been reported that sensitivity to ZEA and its metabolites varies in different experimental animals and livestock species. Pigs are most sensitive to ZEA; this is especially the case for prepubescent sows, as this developmental stage is the most susceptible to the influence of ZEA activity [13,14]. Some studies have shown that pigs effectively absorb orally administered ZEA, at a rate of up to 85% [15]. Once ingested, it can be metabolized to form α-zearalenol and β-zearalenol through intestinal tissue and liver circulation [16]. After penetrating the intestinal wall, ZEA enters the bloodstream and spreads throughout the body, which may cause certain diseases [17]. Therefore, the toxic effects of ZEA on the intestinal tract have increasingly received attention from researchers. To date, most of these studies have focused on morphological changes to the intestines (villus height, crypt depth, villus-gland ratio) [18]. Some studies have shown that ZEA is absorbed in the jejunum and ileum and that it causes oxidative stress [19,20]; however, there are few studies on ZEA-induced oxidative stress in the duodenum of weaned piglets.

The Kelch-like erythroid cell-derived protein with CNC homology-associated protein 1–nuclear factor erythroid 2-related factor 2 (Keap1-Nrf2) signaling pathway is a redox-sensitive signal transduction pathway that is a particularly important anti-oxidative-stress mechanism in the digestive system [21]. Studies have confirmed more than two hundred protective genes downstream of the Keap1-Nrf2 signaling pathway, including genes encoding antioxidant enzyme proteins and phase II detoxification enzymes, all of which play an important role in maintaining tissue homeostasis [22]. Therefore, this signaling pathway has become an important area of focus in the field of oxidative stress research. Oxidative damage *in vivo* can activate signaling pathways through the transcription of these cell-protection genes.

Although ZEA has been thoroughly studied, the mechanism by which its oxidative stress-related toxicity is mediated is unknown, as is the ZEA-mediated Keap1-Nrf2 signaling pathway. Although observable changes are negligible under physiological conditions, any disruption to organ and tissue function by ZEA may impair homeostasis and thus affect the healthy growth of the body. The purpose of this study was to explore the mechanism of ZEA-induced duodenal oxidative stress in post-weaning gilts in order to find a new theoretical basis and potential avenues for the treatment and prevention of ZEA-induced intestinal oxidative stress in post-weaning gilts.

## MATERIALS AND METHODS

All animals used in the present study were cared for in accordance with the guidelines for the care and use of laboratory animals set by the Animal Nutrition Research Institute of Shandong Agricultural University (Tai’an, Shandong, China) and the Ministry of Agriculture of China (Beijing, China).

### Preparation of zearalenone-zontaminated diets

Purified ZEA (Fermentek Ltd., Jerusalem, Israel) was first dissolved in acetic ether, and then poured onto talcum powder. The ZEA premix was prepared by blending ZEA-contaminated talcum powder with ZEA-free corn, which was subsequently mixed with a corn–soybean meal to prepare ZEA-contaminated diets 0 (Control), 0.5 (ZEA0.5), 1.0 (ZEA1.0), and 1.5 (ZEA1.5) mg/kg. We chose the concentration range of 0.5 to 1.5 mg ZEA/kg considering the study of Jiang et al [23,24] and the feeding situation in China. The maximum allowable ZEA concentration in the diet of post-weaning gilts is 0.5 mg/kg in Chinese Feeding Standard. However, swine diets occasionally contain 1 to 1.5 mg/kg ZEA [4]. In the present study, all diets were prepared in one batch, and then stored in covered containers before feeding. A composite sample of each experimental diet was prepared to determine the concentration of ZEA and other mycotoxins at the start and end of the feeding experiment. Zearalenone was analyzed by immunoaffinity column chromatography. Aflatoxin (AFL) was measured by liquid chromatography-fluorescence detection, while deoxynivalenol (DON) was measured by liquid chromatography combined with UV detection. The detection limit was 1.0 μg/kg, 0.1 mg/kg, and 0.1 mg/kg for AFL, ZEA, and DON, respectively (sum of 3-acetyl DON, 15-acetyl DON, and nivalenol). The ZEA concentration was <0.1, 0.52±0.07, 1.04±0.03, and 1.51± 0.13 mg/kg in the control, ZEA0.5, ZEA1.0, and ZEA1.5 respectively. Aflatoxin and DON were not detected in any of the diets.

### Experimental design, animals, and management

Forty post-weaning gilts (Landrace×Yorkshire×Duroc) with an average body weight of 14.01±0.86 kg were randomly allocated to four experimental groups after 10 d of acclimation. The four groups of piglets were then randomly assigned to the four experimental diets as described above and fed the respective diet for 35 d. The treatments were arranged as a completely randomized design. All piglets were individually housed in cages in a temperature-controlled room at the Animal Husbandry Science and Technology Park of Shandong Agricultural University. The diets (Table 1) used in the study were isocaloric and isonitrogenous, with the only difference being the ZEA concentration. The concentration of all nutrients met or exceeded the minimum requirements established by the NRC [26]. Representative samples of each diet were collected at the beginning and end of the experiment for nutrient analyses using the AOAC methods [27].

### Sample collection and preparation

On the last day of the feeding experiment, the piglets were euthanized after fasting for 12 h. The duodenum was immediately isolated from the body, and then dissected into four portions. Three portions were immediately frozen in liquid nitrogen and stored at −80°C for the subsequent analysis of antioxidant enzyme activity, gene expression, and western blotting. The remaining portion was quickly fixed in Bouin’s solution for 24 to 48 h using graded alcohol concentrations for hematoxylin and eosin staining and immunohistochemical analysis.

### Determination of antioxidant enzyme activity

The frozen duodenum samples were thawed, rinsed with ice-cold deionized water, and dried using a filter paper. The samples were then homogenized with 0.02 mmol/L Tris HCl (pH 7.4) at a ratio of 1:10 (mg/mL), followed by centrifugation (10,000×g) at 4°C for 15 min. The supernatant was collected to analyze the total superoxide dismutase (T-SOD) and glutathione peroxidase (GSH-PX) activities, and malondialdehyde (MDA) concentration using the methods described by Jiang et al [28] with SOD A001-1, GSH-PX A005, and MDA A003 Assay Kits, respectively (Nanjing Jiancheng Bioengineering Institute, Nanjing, China). The protein concentration was also determined using the method of Bradford [29] with a protein assay kit (A045; Nanjing Jiancheng Bioengineering Institute, China).

### Immunohistochemical analysis for integrated optical density of Nrf2 and GPX1

For this analysis, the duodenum samples stored in Bouin’s solution were sliced into 5-μm sections using a Leica microtome (RM 2235; Leica Biosystems Nussloch GmbH, Nussloch, Germany). The sections were then processed by immobilization on poly-l-lysine-coated glass slides, drying overnight at 37°C, dewaxing, rehydration, and antigen retrieval in sodium citrate buffer (0.01 mol/L, pH 6.0) using a microwave unit for 20 min at full power, which was followed by washing three times (5 min per washing) with phosphate buffered solution (0.01 mol/L, pH 7.2). The subsequent sample processing for immunohistochemical analysis was the same as that described by Zhou et al [30].

To evaluate the extent of staining and quantity of the target antigen in Nrf2- and glutathione peroxidase 1 (GPX1)-positive cells, images were randomly captured using a microscopic camera system at 100× magnification, which was then analyzed using image analysis software (Image-Pro Plus 6.0; Media Cybernetics, Inc., Rockville, MD, USA) to obtain the total cross-sectional integrated optical density (IOD) [31]. Six stained samples randomly selected from 10 piglets per treatment were used in this analysis.

### Quantification of Keap1, Nrf2, GPX1, NQO1, HO1, GCLM, GCLC, and GAPDH mRNA expression using quantitative real-time polymerase chain reaction

The total RNA was extracted from the duodenum samples stored at −80°C using RNAiso Plus (D9108B; Applied TaKaRa, DaLian, China) according to the manufacturer’s instructions. The purity and concentration of the RNA were assessed using an Eppendorf Biophotometer (RS323C; Eppendorf Aktien Gesellschaft, Hamburg, Germany) at an absorbance ratio of 260:280 nm (a range of 1.8 to 2.0 indicates pure RNA sample). The RNA integrity was verified by agarose gel electrophoresis. The total RNA was reverse transcribed to cDNA using a Reverse Transcription System Kit (Prime-Script RT Master Mix, RR036A; Applied TaKaRa, China).

The polymerase chain reaction (PCR) mixture of total volume 20 μL, containing 10 μL of SYBRY Premix Ex Taq II, 0.4 μL of DyeII (SYBRY Premix Ex Taq-TIi RNaseH Plus, DRR420A; Applied TaKaRa, China), 0.4 μL of both forward and reverse primers, and 2 μL of cDNA (<100 ng), was used for the quantitative real-time PCR (qRT-PCR) analysis. The optimized qRT-PCR protocol included an initial denaturation step at 95°C for 30 s, followed by 43 cycles at 95°C for 5 s, 60°C for 34 s, 95°C for 15 s, 60°C for 60 s, and 95°C for 15 s. The qRT-PCRs were conducted in an AB 7500 Real Time PCR System (Applied Biosystems, Foster City, CA, USA). The relative expression level of Keap1, Nrf2, GPX1, quinone oxidoreductase 1 (NQO1), hemeoxygenase 1 (HO1), modifier subunit of glutamate-cysteine ligase (GCLM), catalytic subunit of glutamate-cysteine ligase (GCLC), and glyceraldehyde-3-phosphate dehydrogenase mRNA was calculated using the 2^–^^ΔΔ^^Ct^ method [32]. The analysis was repeated thrice per sample. The sequence and production length of primers are presented in Table 2.

### Nuclear protein extraction and western blot analysis

The total protein was extracted from the duodenum samples stored at −80°C using radioimmunoprecipitation assay lysis buffer supplemented with phenylmethanesulfonyl fluoride (Beyotime Biotechnology, Shanghai, China) according to the manufacturer’s instructions. The samples were incubated on ice for 30 min and the supernatant was collected by centrifugation (12,500×g) at 4°C for 10 min. After protein quantification using the Bicinchoninic Acid Protein Assay Kit (Tiangen Biotech Co., Ltd., Beijing, China), the solution containing 50 μg of total protein was loaded onto a sodium dodecyl sulfate polyacrylamide gel and subjected to electrophoresis. The proteins were transferred on to 0.22-μm polyvinylidene difluoride (PVDF) membranes (Solarbio, Beijing, China). After blocking the PVDF membranes in 5% skim milk for 2 h, the blots were washed for 30 min with Tris-buffered saline containing 0.1% Tween 20 (TBST: 20 mM Tris, pH 7.5; 150 mM NaCl; and 0.1% Tween-20) and incubated overnight at 4°C with the following primary antibodies: Nrf2 (1:1,000; sc-722, Santa Cruz Biotechnology, Shanghai, China), GPX1 (1:1,000; sc-22145, Santa Cruz Biotechnology, China), Keap1 (1:500; ab218815, Abcam, Shanghai, China), HO1 (1:10,000; ab68477, Abcam, China), NQO1 (1:10,000; ab80588, Abcam, China), GCLM (1:5,000; ab124827, Abcam, China), GCLC (1:10,000; ab190685, Abcam, China), and β-actin (1:2,000; Beyotime Biotechnology, China). The membranes were then washed in TBST for 30 min and incubated again at 37°C with anti-rabbit immunoglobulin G (IgG) antibody (1:5,000; Beyotime Biotechnology, China), and anti-mouse IgG (1:5,000; Beyotime Biotechnology, China) for 1.5 h. Following washing with TBST for 30 min, the membranes were immersed in a high-sensitivity luminescence reagent (BeyoECL Plus; Beyotime Biotechnology, China), and then exposed to film using a Fusion FX imaging system and analyzed using a FusionCapt Advance FX7 software (Beijing Oriental Science and Technology Development Co., Ltd., Beijing, China). The concentration of proteins was determined using Image-Pro Plus 6.0 (Media Cybernetics, Inc., USA).

### Statistical analysis

All data were subjected to one-way analysis of variance using the generalized linear model procedure of SAS 9.2 (SAS Inst. Inc., Cary, NC, USA). The data were initially analyzed as a completely randomized design, with treatment as the fixed effect and individual piglet as random factor. Orthogonal polynomial contrasts were used to determine linear and quadratic responses to the dietary ZEA concentrations. The significance of differences among treatments was tested using Duncan’s multiple range tests and significance was declared at p<0.05.

## RESULTS

### Effects of ZEA on antioxidant enzyme activity of the duodenum

The activities of T-SOD and GSH-Px in the duodenum decreased linearly (p<0.05) and quadratically (p<0.05) as the dietary ZEA concentration increased from 0 to 1.5 mg/kg (Table 3). The MDA concentration in the duodenum was significant increased (p<0.05) in the ZEA1.0 and ZEA1.5 group. The lowest (p<0.05) T-SOD and GSH-Px activities were observed in piglets in the ZEA1.5 group, and the highest MDA concentration (p<0.05) was observed the ZEA1.0 group.

### Effects of ZEA on Nrf2 and GPX1 immunoreactivities in the duodenum

**Figure 1:**
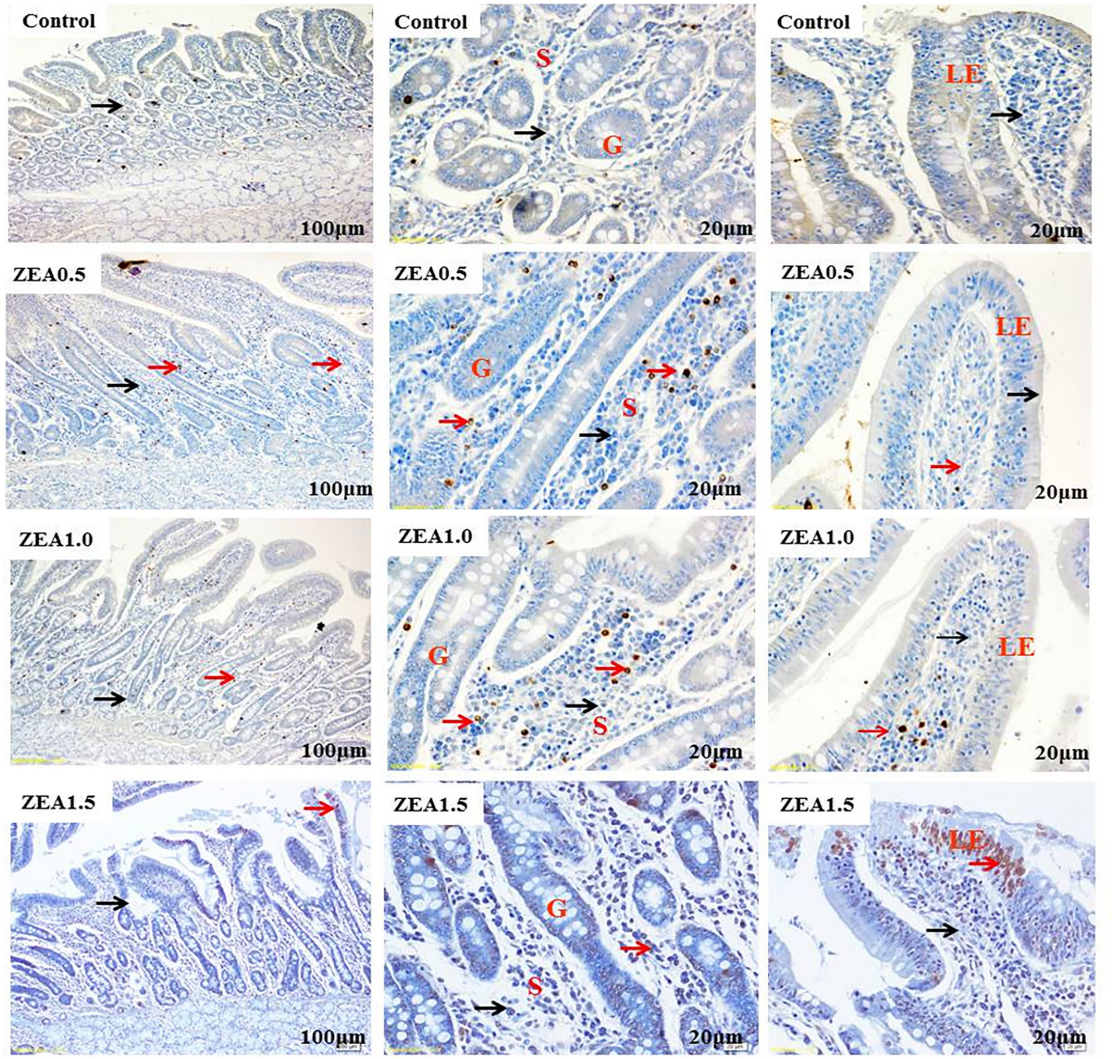
Effects of zearalenone (ZEA) on the localization of nuclear factor erythroid 2-related factor 2 (Nrf2) in the duodenum of post-weaning gilts. All four treatments are shown as follows: Control (A1, A2, and A3); ZEA0.5 treatment (B1, B2, and B3); ZEA1.0 treatment (C1, C2, and C3); and ZEA1.5 treatment (D1, D2, and D3). Control, ZEA0.5, ZEA1.0, and ZEA1.5 represent the control diet with an addition of 0, 0.5, 1.0, and 1.5 mg/kg ZEA, and with analyzed ZEA concentrations of <0.1, 0.52±0.07, 1.04±0.03, and 1.51±0.13 mg/kg, respectively. LE is intestinal villus epithelium, G is intestinal gland, and S is lamina propria. Red arrows represent immunoreactive cells of Nrf2 and blue arrows represent immune-negative cells of Nrf2. Scale bars: approximately 20 μm for A2, B2, C2, D2, A3, B3, C3, D3, and 100 μm for A1, B1, C1, D1.

**Figure 2:**
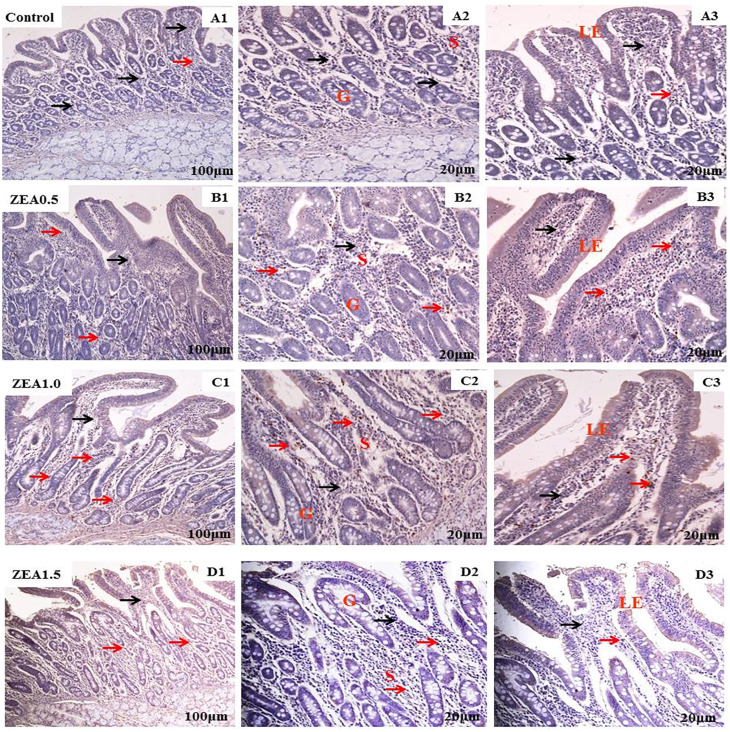
Effects of zearalenone (ZEA) on the localization of glutathione peroxidase 1 (GPX1) in the duodenum of post-weaning gilts. All four treatments are shown as follows: Control (A1, A2, and A3); ZEA0.5 treatment (B1, B2, and B3); ZEA1.0 treatment (C1, C2, and C3); and ZEA1.5 treatment (D1, D2, and D3). Control, ZEA0.5, ZEA1.0, and ZEA1.5 represent the control diet with an addition of 0, 0.5, 1.0, and 1.5 mg/kg ZEA, and with analyzed ZEA concentrations of <0.1, 0.52±0.07, 1.04±0.03, and 1.51±0.13 mg/kg, respectively. LE is intestinal villus epithelium, G is intestinal gland, and S is lamina propria. Red arrows represent immunoreactive cells of GPX1 and blue arrows represent immune-negative cells of GPX1. Scale bars: approximately 20 μm for A2, B2, C2, D2, A3, B3, C3, D3, and 100 μm for A1, B1, C1, D1.

Immunohistochemical analysis showed that the Nrf2 and GPX1 immunoreactive substances were mainly localized in the lamina propria (S) around the intestine gland (G), whereas negative or faint Nrf2 and GPX1 immunoreactivity was observed in most of the intestinal villus epithelium of the duodenum (Nrf2, Figure 1; GPX1, Figure 2). Staining revealed a light yellow immunoreactive substance indicating Nrf2 and GPX1 in the control (A). The localization pattern of positive substances found in the ZEA-treated piglets was comparable with that in the control (red arrows). However, the positive reactions of Nrf2 and GPX1 were linearly (p< 0.05) and quadratically (p<0.05) enhanced (A1-B1-C1-D1) compared with those of the control by increased ZEA concentrations (Table 4), as indicated by the IOD values.

### Effects of ZEA on the relative mRNA expressions of Keap1, Nrf2, GPX1, NQO1, HO1, GCLM, and GCLC

The relative duodenal mRNA expressions of Nrf2 and GPX1 increased both linearly and quadratically (p<0.05) in each of the treatment groups (Table 5). However, the relative mRNA expressions of Keap1 and GCLC decreased (p<0.05) linearly and quadratically with the increase in ZEA concentration. Compared to the control group, piglets in each of the treatment groups presented low (p<0.05) relative mRNA Keap1 expression and high (p<0.05) relative Nrf2 and GPX1 expressions; the relative mRNA expressions of NQO1 and HO1 were high (p<0.05) at ZEA concentrations of 0.5 mg/kg and 1.0 mg/kg, respectively.

### Effects of ZEA on the relative expression of Keap1, Nrf2, GPX1, NQO1, HO1, GCLM, and GCLC proteins

**Figure 3:**
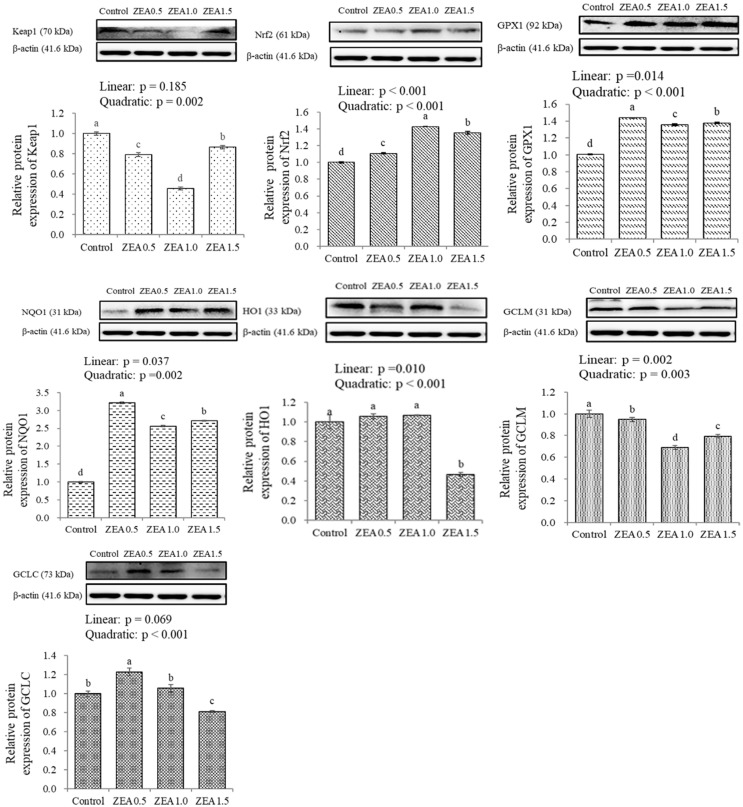
Effects of zearalenone on the protein expressions of Keap1, Nrf2, GPX1, NQO1, HO1, GCLM, and GCLC in the duodenum of post-weaning gilts. Control, ZEA0.5, ZEA1.0, and ZEA1.5 represent the control diet with an addition of 0, 0.5, 1.0, and 1.5 mg/kg ZEA, and with analyzed ZEA concentrations of <0.1, 0.52±0.07, 1.04±0.03, and 1.51±0.13 mg/kg, respectively. Nrf2, nuclear factor erythroid 2-related factor 2; Keap1, Kelch-like ECH-associated protein1; GPX1, glutathione peroxidase 1; NQO1, quinone oxidoreductase 1; HO1, hemeoxygenase 1; GCLM, modifier subunit of glutamate-cysteine ligase; GCLC, catalytic subunit of glutamate-cysteine ligase. ^a–d^ Different letters indicate significant differences between values (p<0.05).

Western blot analysis revealed positive bands of appropriate sizes for all the studied proteins (Keap1, Nrf2, GPX1, NQO1, HO1, GCLM, GCLC, and β-actin; Figure 3). The relative abundances of Nrf2, GPX1, and NQO1 increased (p<0.05) linearly and quadratically with the increase in ZEA levels. On the other hand, the relative abundances of GCLM decreased (p<0.05) linearly and quadratically, and the relative abundance of Keap1 decreased (p<0.05) quadratically with increasing ZEA levels. While the expression of most individual proteins varied, but not significantly, over the different dietary ZEA concentrations, the abundance of Keap1 and GCLM decreased (p<0.05) and those of Nrf2, GPX1, and NQO1 increased (p<0.05) in all treatment groups. However, the relative abundance of HO1 decreased (p<0.05) only in the ZEA1.5 group, and the relative abundance of GCLC increased (p<0.05) in the ZEA0.5 group and decreased (p<0.05) in the ZEA1.5 group of piglets.

## DISCUSSION

A growing number of studies show that ZEA can interfere with the antioxidant-related mechanisms of cells; affect the levels and activities of MDA, SOD, GSH-Px, and other antioxidant enzymes in different cell types; and induce oxidative stress [33,34]. In this study, we hypothesized that ZEA induces oxidative stress in the duodenum of post-weaning gilts. In order to demonstrate this, we evaluated the level of MDA (a product of lipid peroxidation of ROS in duodenal cells), along with changes in the activities of T-SOD and GSH-Px as three indicators of oxidative stress. The experimental data support our conjecture by showing that ZEA significantly reduces the activities of the antioxidant enzymes T-SOD and GSH-Px, increases the MDA content, and causes oxidative stress in the duodenum. Much previous research on the oxidative stress caused by ZEA has revealed that different concentrations of ZEA can significantly increase the MDA levels of human hepatoma cells [35] and Caco-2 cells [36], thus causing oxidative stress. Ren et al [37,38] found that ZEA can reduce the antioxidant enzyme activity of the spleen in mice and increase MDA content. It has been found that dietary contamination with ZEA (1.0 mg/kg) can significantly reduce SOD and GSH-Px activities and significantly increase MDA content in the livers of weaned piglets [39]. Our previous studies have shown that ZEA can significantly reduce T-SOD and GSH-Px activities and increase MDA content in the jejunum and ileum, which further supports our conjecture that ZEA can induce intestinal oxidative stress in weaned piglets [20,40]. More significantly, we found increased GPX1 immunoreactivity in the lamina propria of the duodenal gland of post-weaning gilts fed a ZEA-contaminated diet; this was confirmed by the deep staining that indicated the presence of this protein. This finding is consistent with the significant increase in relative mRNA and protein expressions of the antioxidant gene GPX1. We believe that the increase in GPX1 expression may be a protective mechanism by which ZEA-induced oxidative stress is resisted in the duodenum of post-weaning gilts. In order to better study the mechanism of ZEA-induced oxidative stress, we conducted a more in-depth experiment.

In recent years, the Keap1-Nrf2 signaling pathway has become recognized as one of the most important mechanisms of cellular antioxidant defense, and has therefore been the focus of much research [41,42]. A key transcription factor of this signaling pathway that is responsive to anti-oxidative stress is Nrf2, which is highly expressed in the gastrointestinal tract [21]. Activated Nrf2 can bind with the antioxidant response element (ARE) to form the Nrf2/ARE signal transduction pathway and induce the transcription of downstream antioxidant genes (e.g., *GPX1*) and the phase II detoxification enzyme genes *HO1*, *NQO1*, *GCLM*, and *GCLC* [43]. Under normal physiological conditions, Keap1 combines with Nrf2 in the cytoplasm to form a dimer. In this way, Keap1 negatively regulates Nrf2, inhibiting its activity and thereby maintaining cell homeostasis. When exposed to poisons or certain stimuli, the Keap1-Nrf2 dimer is automatically separated, and Nrf2 is activated and transferred to the nucleus to bind to ARE, thereby activating the expression of antioxidant enzymes and phase II detoxification enzyme genes [44,45]. Therefore, the decrease of antioxidant enzyme activity and the increase of GPX1 expression in intestinal oxidative stress induced by ZEA may be the result of an oxidative stress signal induced by the Keap1-Nrf2 signaling pathway and the regulation of antioxidant enzyme expression by the transcription factor Nrf2.

In this study, we found enhanced Nrf2 immunoreactivity in the duodenal gland of post-weaning gilts fed a ZEA-contaminated diet. Deepened staining indicated a positive reaction, and significant increases were observed in the relative mRNA expression and protein level of Nrf2 in the duodenum. These results indicate that Nrf2 is activated under oxidative stress and may protect the duodenum from the ensuing effects. We further found that the relative mRNA and protein expression of Keap1 decreased significantly, and the relative protein abundance of NQO1 increased significantly in the ZEA experimental groups. Interestingly, while Keap1 was significantly reduced at both the mRNA and protein levels in the duodenum, the relative mRNA and protein expression of NQO1, HO1, and GCLM were significantly increased in the jejunum and ileum with ZEA-induced oxidative stress [20,40]. These results suggest that ZEA induces more obvious oxidative stress in the jejunum and ileum Moreover, the expression of Nrf2 in the Keap1-Nrf2 signaling pathway also increased, confirmed by increases in the optical density of Nrf2 immunoreactive substances. These differences may be due to the different digestion and absorption capacity of ZEA in the duodenum, jejunum, and ileum or to different tolerance of ZEA in the duodenum, jejunum, and ileum. Most importantly, however, when post-weaning gilts are fed ZEA-contaminated diets, the Keap1-Nrf2 signaling pathway is activated and may play an important role in promoting the health of the duodenum, jejunum, and ileum [20,40]. These findings further indicate that the Keap1-Nrf2 signaling pathway in the duodenum of post-weaning gilts fed ZEA-contaminated diets is activated and may play an important protective role. Studies have found that increased levels of Nrf2 in mouse hearts and upregulation of antioxidant enzymes regulated by the Keap1-Nrf2 signaling pathway can offset isoproterenol-induced oxidative stress [46]. It has been reported that the levels of HO1, NQO1, and Nrf2 significantly increase in the kidneys of rats subjected to lipopolysaccharide-stimulated oxidative stress [47]. The same study found that stimulating the production of HO1 and Nrf2 proteins can reduce oxidative damage to the livers of mice and regulate the activity of antioxidant enzymes [48]. Similar to the results of this study, the previous studies mentioned further show that the Keap1-Nrf2 signaling pathway is activated in response to oxidative stress, and protects the body by resisting it. This shows that ZEA can induce intestinal oxidative stress, and the transcription factor Nrf2 in the Keap1-Nrf2 signaling pathway can be used as a potential therapeutic target to resist the oxidative damage caused by ZEA. Due to the complex *in vivo* environment of post-weaning gilts, determining the role of the Keap1-Nrf2 signaling pathway in ZEA-induced duodenal oxidative stress requires verification through further experiments.

In summary, our study shows that ZEA reduces the antioxidant enzyme activity of the duodenum of post-weaning gilts and induces duodenal oxidative stress. The increased expression of Nrf2, GPX1, HO1, NQO1 and the significant decrease of Keap1 expression indicate that the Keap1-Nrf2 signaling pathway is activated and may play an important role under conditions of oxidative stress. The protective effect of the Keap1-Nrf2 signaling pathway in ZEA-induced intestinal oxidative stress in piglets provides new possibilities for solving the toxicity of ZEA in the intestinal and digestive systems and also provides new strategies for the healthy production of animals. In order to better study the role of the Keap1-Nrf2 signaling pathway in ZEA-induced intestinal oxidative stress, *in vitro* cell testing is the goal of our next study.

## Figures and Tables

**Table 1:** Ingredients and nutrient levels of the basal diet (air dry basis, %)

Items	Composition
Ingredients (%)	
Corn	64.5
Whey powder	5.0
Soybean meal	23.0
Fish meal	5.0
L-lysine HCl	0.2
CaHPO_4_	0.7
Pulverized limestone	0.3
NaCl	0.3
Premix^1)^	1.0
Nutrients^2)^	
Digestible energy (MJ/kg)	13.81
Crude protein (%)	19.82
Calcium (%)	0.70
Total phosphorus (%)	0.64
Lysine (%)	1.22
Sulfur amino acid (%)	0.65
Threonine (%)	0.75
Trptophan (%)	0.22

1) Premix provided the following per kilogram of diet: 3,300 IU vitamin A, 330 IU vitamin D_3_, 24 IU vitamin E, 0.75 mg vitamin K_3_, 1.50 mg vitamin B_1_, 5.25 mg vitamin B_2_, 0.02625 mg vitamin B_12_, 15.00 mg pantothenate, 22.50 mg niacin, 0.075 mg biotin, 0.45 mg folic acid, 6.00 mg Mn, 150 mg Fe, 150 mg Zn, 9.00 mg Cu, 0.21 mg I, and 0.45 mg Se.

2) Digestible energy was obtained from digestion experiment [25], whereas the other nutrient contents were calculated values.

**Table 2:** Sequence of primers for real-time polymerase chain reaction

Target gene	Accession No.	Primer Sequence (5′ to 3′)^1)^	Product size (bp)
*Nrf2*	RA_011763	F: GAAAGCCCAGTCTTCATTGC R: TTGGAACCGTGCTAGTCTCA	190
*GPX1*	NC_010444.3	F: GGCACAACGGTGCGGGACTA R: AGGCGAAGAGCGGGTGAGCA	159
*Keap1*	NM_001114671.1	F: GCCTCATCGAGTTCGCTTAC R: CACGGACCACACTGTCAATC	105
*HO1*	NM_001004027.1	F: GCTGAGAATGCCGAGTTCAT R: TGTAGACCGGGTTCTCCTTG	142
*NQO1*	NM_ 001159613.1	F: TGAATTACATCTCTGTGGTTTA R: AGAATGACACTCATATTAGGCG	171
*GCLM*	CV868255.1	F: ACAATACAACGGTTCAGGTGAGT R: GCCTGTAAAATGTGTCATTGAGG	122
*GCLC*	CV864761.1	F: ATGGGCTGGGAACAAGATGT R: GTAACAGGTCTGCATCCTCATC	119
*GAPDH*	NM_001206359.1	F: ATGGTGAAGGTCGGAGTGAA R:CGTGGGTGGAATCATACTGG	154

*Nrf2*, nuclear factor erythroid 2-related factor 2; *Keap1*, Kelch-like ECH-associated protein1; *GPX1*, glutathione peroxidase 1; *HO1*, hemeoxygenase 1; *NQO1*, quinone oxidoreductase 1; *GCLM*, modifier subunit of glutamate-cysteine ligase; *GCLC*, catalytic subunit of glutamate-cysteine ligase; *GAPDH*, glyceraldehyde-3-phosphate dehydrogenase.

1) F, forward primer; R, reverse primer.

**Table 3:** Effects of zearalenone on antioxidant capacity in the duodenum of post-weaning gilts

Items	Control^1)^	ZEA0.5^1)^	ZEA1.0^1)^	ZEA1.5^1)^	SEM	p-values		

						Treatment	Linear	Quadratic
T-SOD (U/mg prot)	19.30^a^	13.14^b^	12.05^c^	11.37^d^	0.109	<0.001	<0.001	<0.001
GSH-Px (U/mL)	165.89^a^	136.53^b^	122.73^c^	112.29^d^	0.845	<0.001	<0.001	<0.001
MDA (nmol/mg prot)	4.62^c^	4.55^c^	6.54^a^	4.99^b^	0.048	<0.001	0.106	0.045

SEM, standard error of the mean; T-SOD, total superoxide dismutase; GSH-PX, glutathione peroxidase; MDA, malondialdehyde.

1) Control, ZEA0.5, ZEA1.0, and ZEA1.5 represent the control diet with an addition of 0, 0.5, 1.0, and 1.5 mg/kg ZEA, and with analyzed ZEA concentrations of <0.1, 0.52±0.07, 1.04±0.03, and 1.51±0.13 mg/kg, respectively.

a–d Values within a row with the different letters mean significantly different (p<0.05).

**Table 4:** Effects of zearalenone on the immunoreactive intergrated optic density of Nrf2 and GPX1 in the duodenum of post-weaning gilts (×10^3^)

Items	Control^1)^	ZEA0.5^1)^	ZEA1.0^1)^	ZEA1.5^1)^	SEM	p-values		

						Treatment	Linear	Quadratic
Nrf2	0.46^b^	0.97^a^	0.92^a^	0.97^a^	0.019	<0.001	<0.001	<0.001
GPX1	0.35^b^	0.67^a^	0.71^a^	0.67^a^	0.008	<0.001	<0.001	<0.001

SEM, standard error of the mean; Nrf2, nuclear factor erythroid 2-related factor 2; GPX1, glutathione peroxidase 1.

1) Control, ZEA0.5, ZEA1.0, and ZEA1.5 represent the control diet with an addition of 0, 0.5, 1.0, and 1.5 mg/kg ZEA, and with analyzed ZEA concentrations of <0.1, 0.52±0.07, 1.04±0.03, and 1.51±0.13 mg/kg, respectively.

a,b Values within a row with the different letters mean significantly different (p<0.05).

**Table 5:** Effects of zearalenone on the relative mRNA expressions of Keap1, Nrf2, GPX1, NQO1, HO1, GCLM, and GCLC in the duodenum of post-weaning gilts

Items	Control^1)^	ZEA0.5^1)^	ZEA1.0^1)^	ZEA1.5^1)^	SEM	p-values		

						Treatment	Linear	Quadratic
Keap1	1.01^a^	1.10^a^	0.63^b^	0.26^c^	0.018	<0.001	0.022	0.005
Nrf2	1.03^b^	1.14^a^	1.14^a^	1.14^a^	0.004	<0.001	<0.001	<0.001
GPX1	1.08^c^	1.21^b^	1.39^a^	1.39^a^	0.014	<0.001	<0.001	<0.001
NQO1	1.00^b^	1.16^a^	0.25^c^	0.19^c^	0.022	<0.001	<0.001	<0.001
HO1	1.00^b^	0.44^c^	1.67^a^	0.26^c^	0.034	<0.001	0.349	0.128
GCLM	1.01^c^	3.19^a^	1.46^b^	1.52^b^	0.067	<0.001	0.903	0.010
GCLC	1.01^a^	0.99^a^	0.55^b^	0.24^c^	0.030	<0.001	<0.001	<0.001

SEM, standard error of the mean; Keap1, Kelch-like ECH-associated protein1; Nrf2, nuclear factor erythroid 2-related factor 2; GPX1, glutathione peroxidase 1; NQO1, quinone oxidoreductase 1; HO1, hemeoxygenase 1; GCLM, modifier subunit of glutamate-cysteine ligase; GCLC, catalytic subunit of glutamate-cysteine ligase.

1) Control, ZEA0.5, ZEA1.0, and ZEA1.5 represent the control diet with an addition of 0, 0.5, 1.0, and 1.5 mg/kg ZEA, and with analyzed ZEA concentrations of <0.1, 0.52±0.07, 1.04±0.03, and 1.51±0.13 mg/kg, respectively.

a–c Values within a row with the different letters mean significantly different (p<0.05).
